# Exploring which symptom should be targeted first in the comorbidity of anxiety and depression among adolescents with nomophobia: insight from a simulation network analysis

**DOI:** 10.3389/fpsyt.2026.1772083

**Published:** 2026-04-13

**Authors:** Jingwen Huang, Peipei Cao, Rongfeng Li, Xiaofan Zhang, Sipu Guo, Shengzhi Liu, Anlun Wan

**Affiliations:** 1 School of Journalism and Communication, Huaqiao University, Xiamen, Beijing, China; 2 Beijing City Research Center, Party School of Beijing Municipal Committee of the Communist Party of China (Beijing Administration Institute), Beijing, China; 3 Shanxi Vocational University of Engineering Science and Technology, Shanxi, Taiyuan, China; 4 Department of Information Management, Peking University, Beijing, China; 5 Peking University Publishing Research Institute, Peking University, Beijing, China; 6 Institute of Developmental Psychology, Faculty of Psychology, Beijing Normal University, Beijing, China; 7 School of Digital Media and Design Arts, Beijing University of Posts and Telecommunications, Beijing, China; 8 School of Journalism and Communication, Beijing Normal University, Beijing, China

**Keywords:** adolescent mental health, anxiety, depression, network analysis, nomophobia, targeted intervention

## Abstract

**Objectives:**

To identify central and bridge symptoms linking anxiety and depression and to determine intervention‐priority symptoms using simulation in Chinese college students with nomophobia.

**Methods:**

A cross-sectional online survey recruited 1, 638 college students in China who met at least mild criteria for nomophobia. Depressive and anxiety symptoms were assessed with the Patient Health Questionnaire-9 (PHQ-9) and Generalized Anxiety Disorder-7 (GAD-7). Ising networks were estimated for depression, anxiety, and their comorbidity. Expected influence and bridge expected influence were computed, and accuracy and stability were examined via nonparametric and case-dropping bootstraps. Network Intervention simulated analysis, symptom-specific alleviating and aggravating interventions to rank targets.

**Results:**

Motor agitation/retardation (PHQ-8) and restlessness (GAD-5) were the most central symptoms in the depression and anxiety networks and remained central in the comorbidity network. Irritability (GAD-6) and feeling afraid (GAD-7) showed the highest bridge centrality, linking anxiety with depressive symptoms. NIRA indicated that reducing fatigue/low energy (PHQ-4) and excessive worry (GAD-3) would yield the largest decreases in overall network activation, whereas activating suicidal ideation (PHQ-9) and restlessness (GAD-5) would most strongly aggravate the network. Bootstrap results supported acceptable accuracy and stability (e.g., EI CS-C ≈.67 for depression;.60 for anxiety; bridge EI CS-C ≈.44 in the comorbidity network).

**Conclusions:**

Symptom-level analysis highlights actionable leverage points in nomophobia-related comorbidity. Longitudinal and experimental studies are warranted to confirm causal pathways and validate network-informed treatment sequencing.

## 1 Introduction

Nomophobia, a term derived from the English phrase “No Mobile Phobia” (1), is the state of mind where one feels afraid or anxious of the possibility of separation from mobile phones (2–5). It has been conceptualized as a modern type of phobia resulting from the pervasive integration of mobile information and communication technologies, particularly smartphones, into daily life (6). In recent years, nomophobia has emerged as a growing global concern and has been linked to various psychological and behavioral health issues (7). As stated by Han et al. (8), if individuals treat smartphones as an extension of themselves, they are more likely to experience an emotional attachment to the devices, which in turn increases proximity-seeking behaviors and contributes to nomophobia (8).

Adolescents have consistently been identified as the demographic most susceptible to high levels of nomophobia (9). Due to the particularity of psychological development, adolescents are more likely to form dependence on mobile phones, especially under high dependence on social media. Research has shown a strong association between smartphone addiction and nomophobia in adolescents (10). Symptoms of nomophobia (such as anxiety) can worsen depressive symptoms, which in turn can increase dependence on the phone, creating a vicious circle. Students whose school downturn is due to smartphone overuse tend to show high anxiety or fear if they cannot easily or quickly access phones or digital operations (11). Conversely, nomophobia has also been shown to affect various psychological and physical aspects such as anxiety, stress, and study performance (12). Though it has been observed in the literature so far that there is a two-way connection between nomophobia and mental problems, especially anxiety and depression, the question of how such mental illnesses and nomophobia communicate on the symptom level is yet unknown. Hence, it is critical to fill such a gap to inform early screening, prevention, and symptom-focused intervention in such a population.

### 1.1 The bidirectional relationship between anxiety and nomophobia

Existing studies and diagnostic criteria indicate that nomophobia exhibits characteristics highly similar to anxiety disorders. According to Yildirim and Correia (6), four core dimensions of nomophobia were identified: not being able to communicate, losing connectedness, not being able to access information, and giving up convenience (6). Beyond these dimensions, nomophobia also manifests hallmark features of anxiety disorders: anticipatory worry, avoidance symptomatology, and physiological hyperarousal, such as increased heart rate, sweating, and nervousness when mobile connectivity is threatened (12, 13). These manifestations resonate strongly with the diagnostic criteria outlined in the Diagnostic and Statistical Manual of Mental Disorders, Fifth Edition, Text Revision (DSM-5-TR; 14). Conceptual overlap has therefore resulted in researchers questioning whether anxiety precedes nomophobia, occurs concurrently, or emerges as a consequence of it.

In light of such conceptual overlap, a growing body of empirical work has sought to examine the association between nomophobia and anxiety. Cumulative findings consistently report a robust positive association between the two constructs (15). In addition, higher anxiety has been linked to a greater likelihood or severity of nomophobia in survey datasets (16). Within educational settings, nomophobia has been associated with heightened stress and anxiety, further handicapping students’ academic performance and learning outcomes (17).

Survey-based evidence provides further substantiation. In one large-scale investigation, about 26.5% of the respondents in one study on nomophobia indicated they had anxiety problems or were on anxiety-related medication (18). This proportion highlights the notable intersection between nomophobia and anxiety. Additional studies demonstrate that greater anxiety was also associated with an increased risk for severe nomophobia (19). Parallel findings were reported by Alodhialah et al. (20), who also reported that elevated nomophobia scores are strongly related to elevated psychological distress, i.e., anxiety (20). Similarly, Bernabe-Mateo et al. (21) confirmed that individuals with high anxiety are more predisposed to acquire nomophobia, further lending evidence to anxiety as a risk factor (21).

These findings not only substantiate the comorbidity of nomophobia and anxiety but also imply that anxiety may play an active role in the onset and exacerbation of nomophobia. The convergence of results highlights the need to consider anxiety-specific mechanisms when theorizing nomophobia, however, much of the current evidence remains correlational.

### 1.2 Comorbidity of anxiety and depression in nomophobia

Cross-sectional studies robustly indicate that individuals with high nomophobia scores exhibit higher levels of depression, anxiety, and stress (16, 22). Similarly, Gnardellis et al. (23) observed subjects with severe nomophobia also presenting elevated degrees of negative emotional states on all subscales of the DASS (Depression, Anxiety, and Stress Scale), including 40.6% presenting depressive symptoms, 73.7% experiencing anxiety symptoms, and 32.7% facing stress. Additionally, individuals presenting heightened depressive and anxiety symptoms were observed to check their mobiles more often and display more compulsive mobile phone use during daily activities (23). However, such associations remain correlational and do not establish directionality or causality.

Longitudinal evidence further substantiates the comorbid relationship between nomophobia and internalizing symptoms. Caba-Machado et al. (9) conducted a two-wave study among adolescents with a six-month follow-up, demonstrating that increases in nomophobia intensity were prospectively associated with subsequent elevations in depression, anxiety, and stress (9). In addition, Daraj et al. (15) conducted a meta-analysis that synthesized data from 16 studies and quantified the correlational relationship between nomophobia and anxiety, reflecting a moderate but consistent association across samples (15).

To sum up, evidence from cross-sectional, longitudinal, and meta-analytic levels consistently demonstrates the comorbidity of anxiety and depression with nomophobia. However, prior studies have largely treated anxiety and depression as global constructs, overlooking how individual symptoms interact with nomophobia and with one another. This limitation highlights the need for approaches capable of mapping and quantifying symptom-level dynamics.

### 1.3 Network analysis and intervention-oriented perspectives

In recent years, network analysis has emerged as a powerful framework in the field of psychopathology, offering novel insights into the complex interplay of mental health symptoms. Rather than conceptualizing mental disorders as latent variables causing observable symptoms, network theory posits that symptoms directly influence one another in dynamic and mutually reinforcing ways. This paradigm shift has led to the growing application of network models in mental health research (24), particularly in identifying key symptoms that sustain or exacerbate psychological conditions.

Despite the increasing use of network analysis to investigate the structure of nomophobia and its associations with anxiety and depression, existing studies have largely focused on identifying central or bridge symptoms within cross-sectional symptom networks. While such approaches deepen our understanding of how nomophobia co-occurs with internalizing symptoms, they seldom extend to exploring the implications of these structures for targeted interventions. Specifically, there remains a critical gap in the literature: few, if any, studies have adopted an intervention-oriented perspective to determine which symptoms should be prioritized for treatment in adolescents experiencing comorbid nomophobia, anxiety, and depression.

To address this gap, this study introduces a simulation-based network intervention approach aimed at identifying the most impactful symptom targets for early intervention. By estimating how symptom activation and deactivation cascade through the network, we aim to determine which nomophobia symptoms would yield the greatest reduction in overall network connectivity and psychological burden when addressed. This approach offers practical implications for personalized intervention strategies and advances the theoretical understanding of symptom-level dynamics in nomophobic adolescents. Ultimately, the core objective of this study is to identify priority symptoms for intervention among adolescents experiencing comorbid nomophobia, thereby informing symptom-specific, efficient, and targeted therapeutic efforts.

## 2 Materials and methods

### 2.1 Procedures and participants

The survey was conducted via Wenjuanxing, a widely used online platform in China (https://www.wjx.cn). A total of 321 participants were excluded because their average response time per item was shorter than 2 seconds, which may indicate insufficient effort responding (25, 26). In contrast, 114 participants were removed due to excessively long response times per item, defined as statistical outliers using the criterion Q3 + 1.5 × IQR (27), corresponding to approximately 10 seconds in the present sample. After these exclusions, 1, 801 participants remained (valid response rate: 80.5%). Among them, 1638 were classified as having at least mild nomophobia, using an average score of each item above 1 as the cutoff (6). Subsequent analyses were conducted based on the 1638 participants (598 males, 36.5%; Age: M = 20.78, SD = 1.60). **Table 1** shows the detailed demographic information of the sample.

**Table 1:** Demographic information.

Variable	N	% of total
Gender		
Male	598	36.5%
Female	1040	63.5%
Only child		
Yes	312	19.0%
No	1326	81.0%
Parental marriage		
Married and living together	1387	84.7%
Married but living apart long-term	82	5.0%
Married, but one or both parents are deceased	57	3.5%
Divorced, neither parent has remarried	63	3.8%
Divorced, at least one parent has remarried	49	3.0%
Parental relationship		
Harmonious relationship, never argue	826	50.4%
Occasionally argue, about 1–2 times per month	749	45.7%
Frequently argue, on nearly half the days each month	53	3.2%
Constantly argue, almost every day	10	0.6%

All participants gave electronic informed consent and were informed of their right to withdraw at any time. The present study was reviewed and approved by the Ethics Committee of Beijing University of Posts and Telecommunications.

### 2.2 Measures

#### 2.2.1 Nomophobia questionnaire

The Nomophobia Questionnaire was used to measure nomophobia, which consists of 16 items divided into four dimensions (28). These included: 1) fear of being unable to access information (e.g., “I would be annoyed if I could not look information up on my smartphone when I wanted to do so.”; Cronbach’s α = .89); 2) fear of losing convenience (e.g., “If I were to run out of credits or hit my monthly data limit, I would panic.”; Cronbach’s α = .88); 3) fear of losing contact (e.g., “I get worried when my family and/or friends can’t reach me because my phone isn’t with me.”; Cronbach’s α = .92); and 4) fear of losing internet connection (e.g., “I feel anxious when being separated from my phone disconnects me from the internet.”; Cronbach’s α = .95). Responses were rated on a seven-point Likert scale (1 = strongly disagree, 7 = strongly agree), with higher scores indicating stronger nomophobia. Based on the average item score, nomophobia severity is classified as follows: none (score = 1), mild (1 < score < 3), moderate (3 ≤ score < 5), and severe (score ≥ 5; 6). In this study, the Cronbach’s α for the full questionnaire was 0.96.

#### 2.2.2 Patient health questionnaire-9

The PHQ-9 was employed to assess the depression symptoms (29). Participants evaluated how often they experienced depressive symptoms (e.g., “depressed mood”, “fatigue”, and “guilt”) over the past two weeks using a four-point Likert scale (0 = Not at all, 3 = almost everyday). Higher total scores reflected greater severity of depression. Based on the total score, depression is classified as follows: none or minimal (0-4), mild (5-9), moderate (10-14), moderately severe (15-19), and severe (20-27). Consistent with previous studies, we adopted a score greater than 9 on the PHQ-9 to identify at least moderate depression among Chinese college students (30, 31). In this study, the Cronbach’s α was 0.91.

#### 2.2.3 Generalized anxiety disorder-7

The GAD-7 was employed to measure the anxiety symptoms (32). Participants evaluated how often they experienced anxiety symptoms (e.g., “anxiousness”, “restlessness”, and “irritability”) over the past two weeks. Responses were rated on a four-point Likert scale (0 = Not at all, 3 = almost everyday), with higher total scores indicating severe anxiety. Based on the total score, anxiety is classified as follows: none or minimal (0-4), mild (5-9), moderate (10-14), and severe (15-21). Existing research indicates that a total score greater than 9 is a reasonable cut-off for identifying generalized anxiety among Chinese college students (31, 33). The Cronbach’s α in this study was 0.93.

#### 2.2.4 Parental marital status and relationship quality

Parental marital status was assessed using a single item (i.e., “What is the current marital status of your parents?”; 1 = Married and living together, 5 = Divorced, at least one parent has remarried). Parental relationship quality was also assessed using a single item (i.e., “How well do your parents communicate with each other?”; 1 = Harmonious relationship, never argue, 5 = Constantly argue, almost every day).

### 2.3 Statistical analysis

All data analyses were conducted using R software (version 4.3.2; 34). First, we conducted descriptive statistics for demographic variables (e.g., age, gender, only-child status, parental marital and relationship status) as well as research variables (e.g., nomophobia, depression, and anxiety). The overall prevalence of depression and anxiety, as well as the prevalence of individual symptoms, was examined. Independent sample t-tests were used to examine gender and only-child status differences across these variables. Correlations were examined between study variables and factors such as age, parental marital status, and relationship quality. To enable simulation-based intervention analyses, which are specifically implemented for Ising networks, we first converted the original continuous symptom scores into binary scores (absent: 0, scores = 0, present: 1, scores > 0). Then, based on these binary scores, we conducted the following network analyses.

#### 2.3.1 Network structure and centrality estimation

Based on the binary data, we estimated the separate Ising networks for depression and anxiety, as well as a comorbidity Ising network for both. The networks were estimated and visualized using the R packages bootnet 1.4.3 and qgraph 1.6.9 (35, 36). The Ising networks were estimated using the estimateNetwork function in the bootnet package with the “IsingFit” algorithm (37). This approach applies nodewise logistic regression, regressing each binary variable on all others, and employs the eLasso procedure with L1 regularization and EBIC model selection (γ = 0.25). The AND-rule was used for edge selection. After estimating network, we could obtain two key parameters: the edge weights and the thresholds. Edge weights (i.e., regression coefficients) represented the relationships between nodes in the network, and thresholds (i.e., intercepts) represented the symptom’s disposition for manifestation. Positive (negative) thresholds denote the symptom’s disposition to be activated (deactivated) when all other symptoms are absent. The Ising networks were visualized using the qgraph 1.6.9 (36); nodes represented the symptoms of depression and anxiety, and edges between nodes represented the relationships. Green (red) edges indicated the positive (negative) relationships, with thicker edges denoting stronger relationships (38).

For the separate depression and anxiety networks, we estimated Expected Influence (EI) using the centrality function from the *qgraph* package (1.6.9; 36, 39). For the combined anxiety–depression network, we estimated both EI and bridge EI. Bridge EI was computed using the bridge function from the *networktools* package (1.6.0; 40). In the comorbidity network, communities were defined *a priori* according to symptom domains, with PHQ-9 symptoms representing depression and GAD-7 symptoms representing anxiety. EI captures the sum of the edge weights connected to a node, indicating its overall influence. Bridge EI captures the sum of connections between a given node and nodes in other symptom communities, reflecting its role in linking distinct clusters (40). Consistent with Tang et al. (41), nodes with standardized centrality values > 1 (i.e., 1 SD above the mean) were highlighted.

To evaluate the potential impact of dichotomizing symptom items on network estimation, we conducted additional analyses based on the original continuous PHQ-9 and GAD-7 item scores. Specifically, three Gaussian graphical models (partial correlation networks) were estimated using the *estimateNetwork* function with the “*EBICglasso*” algorithm (35). The resulting adjacency matrices were extracted and compared with those derived from the corresponding dichotomized Ising networks. A Mantel test was performed to assess the similarity between the continuous and binary network structures (42, 43). A high correlation between the two network matrices would suggest that binarizing the data did not affect the network’s sensitivity.

#### 2.3.2 Network stability and accuracy

The accuracy and stability of all three networks were assessed using the R package bootnet 1.4.3 (35), with all bootstrap analyses set to 1, 000 resamples. First, a nonparametric bootstrapping test was used to compute 95% confidence intervals (CIs) for edge weights; narrower CIs indicated more reliable edge ranking. Second, case-dropping bootstrap procedures were used to measure centrality stability using the correlation stability coefficient (CS-C). The CS-C measures the maximum extent to which the sample size can be reduced while still preserving stability, defined as maintaining, with 95% probability, a correlation of at least 0.7 between the original and subset networks. CS-C values above 0.25, 0.5, and 0.75 indicate acceptable, good, and excellent stability, respectively (44). Additionally, bootstrapped difference tests were performed on edge weights and node centralities.

#### 2.3.3 Simulation of alleviating and aggravating interventions

Simulation-based intervention analyses were performed on all three Ising networks using the NodeIdentifyR algorithm (NIRA) from the R package IsingSampler (45). For each intervention, NIRA systematically adjusted the threshold of a target symptom by ±2 standard deviations of its estimated threshold to simulate alleviating (−2 SD) or aggravating (+2 SD) interventions. Each simulation generated 5, 000 samples using the Metropolis-Hastings algorithm implemented in IsingSampler, ensuring reproducibility with a fixed random seed (seed = 123). One simulation was conducted with all original thresholds, followed by one simulation for each symptom-specific intervention. The effect of each intervention was quantified by the NIRA outcome, defined as the absolute change in the network sum score (sum of all symptom activations) before versus after threshold adjustment. Symptoms producing the largest NIRA outcomes in alleviating interventions were considered effective treatment targets, whereas those with the largest outcomes in aggravating interventions were identified as key prevention targets (45, 46).

## 3 Results

### 3.1 Descriptive statistics, group differences, and correlational analysis

**Table 2** shows descriptive statistics for nomophobia, anxiety, and depression. Using a cutoff score of >9 on the PHQ-9 and GAD-7, 1, 277 participants (78.0%) showed no depression or anxiety symptoms. Among the remaining 361 (22.0%), 143 (8.7%) had only depressive symptoms, 58 (3.5%) had only anxiety symptoms, and 160 (9.8%) had both. Based on the binary scores of symptoms (0 = absent, 1= present), the most common symptoms were “excessive worry” (GAD-3; 66.6%), “anxiousness” (GAD-1; 66.5%), “fatigue” (PHQ-4; 64.0%) and “anhedonia” (PHQ-1; 64.0%).

**Table 2:** Descriptive statistics of the variables.

Variable	M	SD	Skewness	Kurtosis	Binary score
Nomophobia	3.36	1.31	0.23	-0.48	——
Depression	5.76	5.12	0.98	0.90	——
PHQ-1: Anhedonia	1.74	0.72	0.71	0.27	0.64
PHQ-2: Depressed or sad mood	1.46	0.70	1.12	1.79	0.47
PHQ-3: Sleep difficulties	1.30	0.81	0.92	4.22	0.54
PHQ-4: Fatigue	2.25	0.78	0.67	-0.42	0.64
PHQ-5: Appetite changes	1.80	0.78	0.87	0.50	0.56
PHQ-6: Guilt	1.71	0.75	1.16	0.41	0.44
PHQ-7: Concentration difficulties	1.86	0.80	0.92	0.21	0.55
PHQ-8: Motor disturbances	1.73	0.69	1.47	0.33	0.36
PHQ-9: Suicidal ideation	1.60	0.60	2.11	0.67	0.23
Anxiety	5.11	4.33	0.85	0.76	——
GAD-1: Anxiousness	1.63	0.68	0.59	0.78	0.66
GAD-2: Uncontrollable worry	1.74	0.72	0.79	0.27	0.58
GAD-3: Excessive worry	1.46	0.76	0.66	1.79	0.67
GAD-4: Trouble relaxing	1.30	0.76	0.85	4.22	0.57
GAD-5: Restlessness	2.25	0.71	1.02	-0.42	0.48
GAD-6: Irritability	1.80	0.76	0.83	0.50	0.60
GAD-7: Feeling afraid	1.71	0.75	1.08	0.41	0.49

Binary symptom scores were used to estimate the prevalence of each symptom in the sample.

**Supplementary Table S1** showed group differences by gender and only-child status (Yes/No). Males reported higher levels of anxiety (p = <.001, Cohen’s d = 0.18) but lower levels of nomophobia (p <.001, Cohen’s d = -0.26) than females. No gender differences were found in depression (p = .210). In addition, no significant differences were observed between only-child and non-only-child participants across all variables (ps ≥.140). **Supplementary Table S2** displayed the correlations with age, parental marital status, and relationship quality. Age was negatively associated with nomophobia, anxiety, and depression (rs = -.07 to -.15, ps ≤.001). Poorer parental marital status (rs = .09 to.12, ps ≤.001) and relationship quality (rs = .20 to.21, ps ≤.001) were associated with higher depression and anxiety. Additionally, relationship quality was positively correlated with nomophobia (r = .17, p <.001).

### 3.2 Separate network structures and centrality for depression and anxiety

**Figure 1:**
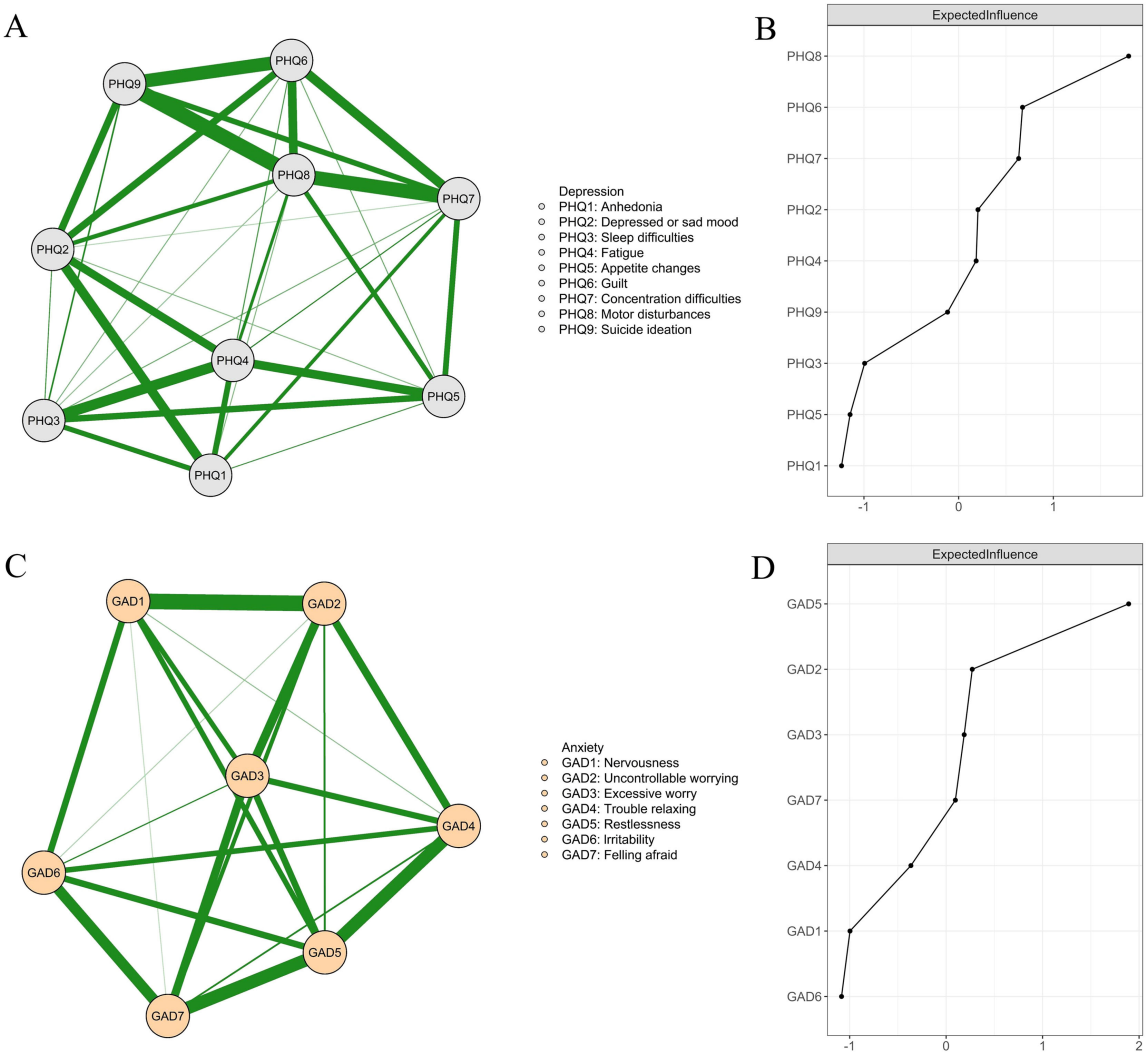
Network structures and node centrality for depression **(A, B)** and anxiety **(C, D)**. All edges in the depression **(A)** and anxiety **(C)** networks were green, indicating positive associations; thicker lines represent stronger connections.

The depression network (**Figure 1A**) contained 33 non-zero edges, all of which were positive (see the edge-weight matrix and thresholds in **Supplementary Table S3**). All nodes in the network showed negative thresholds, indicating that symptoms were most likely to be inactive when others were not activated. The symptom “anhedonia” (PHQ-1; threshold = -1.502) had the threshold value closest to zero, making it the most likely symptom to be activated. As shown in **Figure 1B**, “Motor disturbances” (PHQ-8) had the highest EI value (EI = 1.795), thereby being identified as the central symptom in the network. “Motor disturbances” (PHQ-8) had strong positive connections with other depression symptoms (edge weights ranging from 0.262 to 1.747), particularly with “ suicidal ideation “ (PHQ-9; edge weight = 1.747), “concentration difficulties” (PHQ-7; edge weight = 1.655), and “guilt” (PHQ-6; edge weight = 1.168).

The anxiety network (**Figure 1C**) included 21 non-zero edges, with all being positive (see the edge-weight matrix and thresholds in **Supplementary Table S4**). Since the negative thresholds, all anxiety symptoms tended to be inactive when other symptoms were not activated. The symptom most likely to be activated was “anxiousness” (GAD-1; threshold = -1.746). “Restlessness” (GAD-5) had the highest EI value (EI = 1.892; **Figure 1D**) and was identified as the central symptom. “Restlessness” (GAD-5) was strongly connected to other symptoms (edge weights ranging from 0.595 to 1.713), particularly with “feeling afraid” (GAD-7; edge weight = 1.713) and “trouble relaxing” (GAD-4; edge weight = 1.680).

The additional partial correlation networks of depression and anxiety based on continuous scores are presented in **Supplementary Tables S5**, **S6**, respectively. Mantel tests indicated a high degree of similarity between the two depression network matrices (*r* = .882, *p* = .001) and between the two anxiety network matrices (*r* = .770, *p* = .001). These findings suggest that the binarization procedure adequately preserved the pattern of symptom associations observed in the original continuous data and did not substantially compromise the accuracy or sensitivity of the network estimation.

### 3.3 Depression-anxiety comorbidity network structures and centrality

**Figure 2:**
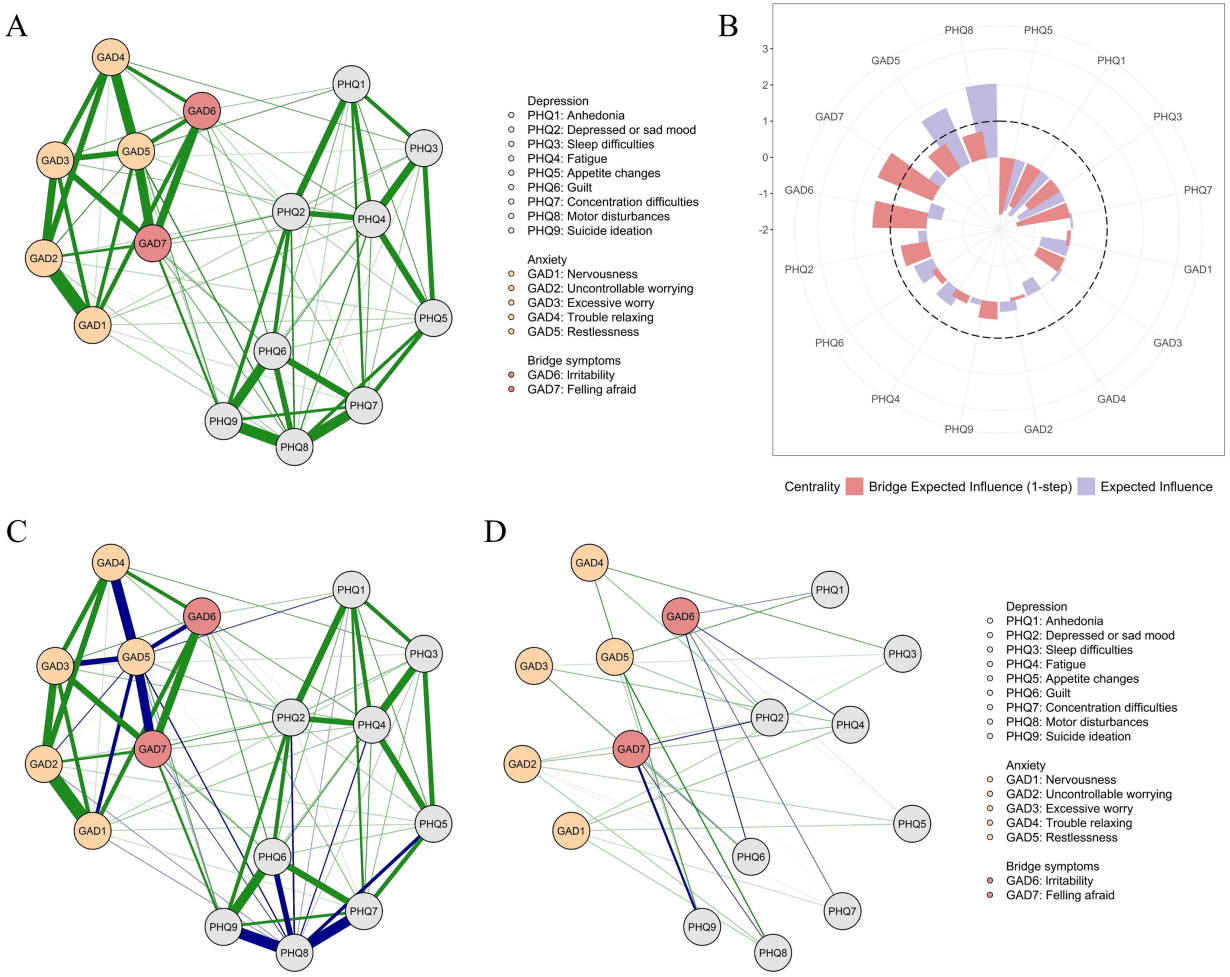
Depression-anxiety comorbidity network structures **(A, C, D)** and node centrality **(B)**. Part **(A)** shows the original network, in which all edges are positive and displayed in green. Part **(C)** highlights the edges connected to the central node (i.e., GAD-5 and PHQ-8), which are shown in darkblue. Part **(D)** displays only the edges between anxiety and depression, with the edges connected to the bridge nodes (i.e., GAD-6 and GAD-7) highlighted in darkblue. In Parts **(A, C, D)**, thicker lines indicate stronger connections.

The depression-anxiety network (**Figure 2A**, **Supplementary Table S7**) contained 87 non-zero edges, all of which were positive. These included 33 edges within depression symptoms, 20 within anxiety symptoms, and 34 connecting depression and anxiety symptoms. All symptoms tended to be inactive when other symptoms were not activated. “Anhedonia” (PHQ-1; threshold = -1.513) and “anxiousness” (GAD-1; threshold = -1.886) were the most likely to be activated. **Figure 2B** displayed the EI and bridge EI values for the nodes. “Motor disturbances” (PHQ-8; EI = 2.036) and “Restlessness” (GAD-5; EI = 1.666) were identified as the two central symptoms in the network. These two nodes were primarily connected to symptoms within the same disorder (**Figure 2C**). As in the depression network, “Motor disturbances” (PHQ-8) was most strongly connected to “ suicidal ideation “ (PHQ-9), “concentration difficulties” (PHQ-7), and “guilt” (PHQ-6; edge weights ranging from 1.005 to 1.599). Similarly, “Restlessness” (GAD-5; EI = 1.666) remained most strongly connected to “trouble relaxing” (GAD-4; edge weight = 1.479) and “feeling afraid” (GAD-7; edge weight = 1.393).

“Feeling afraid” (GAD-7; bridge EI = 1.644) and “Irritability” (GAD-6; bridge EI = 1.493) had the highest bridge EI values and were identified as key bridge symptoms in the comorbid network. These symptoms played important roles in linking depression with anxiety symptoms (**Figure 2D**). “Feeling afraid” (GAD-7) had strong connections with depression symptoms, including “ suicidal ideation “ (PHQ-9), “Motor disturbances” (PHQ-8), “guilt” (PHQ-6), and “depressed or sad mood” (PHQ-2; edge weights ranging from 0.223 to 0.642). Similarly, “Irritability” (GAD-6) had strong connections with depression symptoms such as “concentration difficulties” (PHQ-7), “guilt” (PHQ-6), and “fatigue” (PHQ-4; edge weights ranging from 0.309 to 0.486).

The additional depression–anxiety network based on continuous scores is presented in **Supplementary Table S8**. It was highly correlated with the dichotomized Ising network (*r* = .805, *p* = .001), indicating minimal impact of binarization on network structure.

### 3.4 Network accuracy and stability

Bootstrapped analyses of edge weights (**Supplementary Figure S1**) showed that the edge weights in the depression, anxiety, and comorbidity networks demonstrated acceptable accuracy, with narrow 95% confidence intervals. Case-dropping bootstrap analyses (**Supplementary Figure S2**) further indicated that the centrality values were stable across the depression (EI: CS-C = 0.67), anxiety (EI: CS-C = 0.60), and comorbidity (EI: CS-C = 0.36; bridge EI: CS-C = 0.44) networks. These results suggest the maximum extent to which the sample size can be reduced while preserving the network structure’s stability. Moreover, **Supplementary Figures S3**, **S4** displayed the results of bootstrapped difference tests for edge-weights (**Supplementary Figure S3**) and centrality indices (**Supplementary Figure S4**; e.g., EI and bridge EI). In all three networks, most edge weights and node EIs were significantly different from one another, indicating high uniqueness.

### 3.5 Simulation of alleviating and aggravating interventions

**Figure 3:**
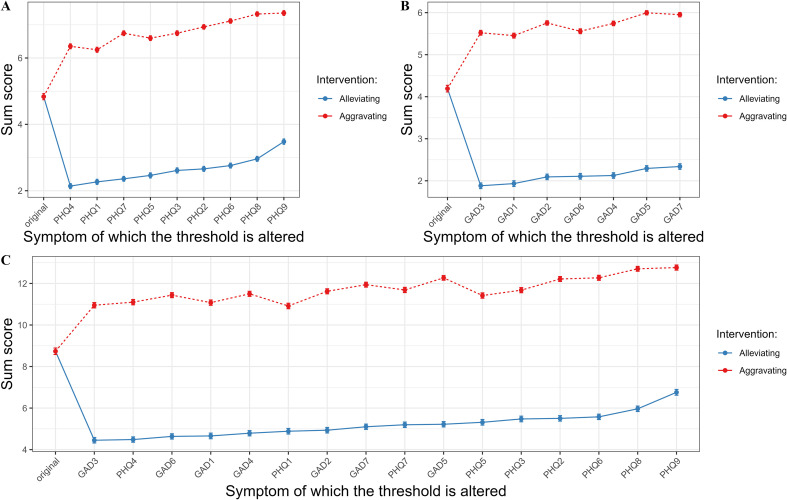
Simulation interventions of the depression **(A)**, anxiety **(B)**, and comorbidity **(C)** networks. Note. Dots represent the network sum scores, while the lines represent the 95% confidence intervals. The symptoms are listed based on the size of the intervention’s effects, along with the original total score before the intervention.

**Figure 3**, **Supplementary Table S9** displayed the results of the simulated interventions for the depression, anxiety, and comorbidity networks. First, the alleviating intervention simulations suggested that targeting “fatigue” (PHQ-4, NIRA = 2.70; **Figure 3A**) and “excessive worry” (GAD-3, NIRA = 2.31; **Figure 3B**) would be the most effective in reducing overall symptom severity for depression and anxiety, respectively. Furthermore, in the comorbidity network, these two symptoms (GAD-3: NIRA = 4.25, PHQ-4: NIRA = 4.28; **Figure 3C**) also emerged as the most effective targets for lowering overall symptom severity. Second, the aggravating intervention simulations suggested that targeting “ suicidal ideation “ (PHQ-9, NIRA = 2.52; **Figure 3A**) and “restlessness” (GAD-5, NIRA = 1.80; **Figure 3B**) would be most effective in increasing overall symptom severity for depression and anxiety, respectively. Moreover, for the comorbidity network, “ suicidal ideation “ (PHQ-9, NIRA = 4.03; **Figure 3C**) was identified as the symptom whose activation most significantly heightened the overall symptom severity.

## 4 Discussion

This study provides a nuanced, symptom-level perspective on the co-occurrence of depression and anxiety in adolescents with nomophobia. Using network analysis and simulation-based Network Intervention Analysis (NIRA), we identified specific symptoms that are most influential and those that bridge the depression-anxiety interface. The psychomotor disturbance symptom of depression (PHQ-8) and the irritability symptom of anxiety (GAD-5) emerged as the most central nodes, indicating they have the strongest overall connections to other symptoms in the combined network. Meanwhile, two anxiety-related symptoms, “fear that something awful might happen” (GAD-7) and “becoming easily annoyed or irritable” (GAD-6), stood out as bridge symptoms linking the depression and anxiety clusters. These bridge symptoms suggest concrete pathways through which anxiety and depressive symptoms co-occur, resonating with the definition of nomophobia as a fear of being without one’s phone (6) and with evidence that nomophobia often presents alongside elevated anxiety and depression (16, 22, 23).

Importantly, the NIRA simulations revealed potential intervention priorities. Reducing “fatigue or low energy” (PHQ-4) or easing “trouble relaxing” (GAD-3) produced the largest simulated decreases in overall symptom activation, suggesting these as optimal targets for alleviation. In contrast, exacerbating “suicidal ideation” (PHQ-9) or increasing “restlessness/irritability” (GAD-5) led to the greatest surge in total network severity, marking them as the most potent aggravating factors. In other words, PHQ-4 and GAD-3 were identified as the most effective points to intervene to reduce symptoms, whereas PHQ-9 and GAD-5 would, if worsened, have the most dramatic impact on the entire symptom network. Notably, these intervention targets were not identical to the most central symptoms, underscoring that network centrality does not necessarily equate to clinical priority. This divergence aligns with findings from recent network analyses (46) and prior methodological cautions (44, 45), which show that the symptoms most tightly connected in a network are not always the ones whose change would yield the largest overall effect. Together, these results extend prior research on nomophobia and its internalizing comorbidities by pinpointing which specific symptoms might drive the well-documented associations between severe nomophobia and heightened depression/anxiety (9, 15, 16).

The pattern of findings can be interpreted through the lens of Attention Control Theory (ACT) (47), which proposes that anxiety consumes cognitive resources and undermines executive control. In the context of nomophobia, pervasive worry about being without one’s smartphone may create a baseline of hypervigilance and distractibility. This chronic anxious arousal can manifest in symptoms like irritability and restlessness, precisely the features we observed as central in the network. ACT would predict that students anxious about phone separation have difficulty disengaging from intrusive thoughts about their phones, leaving fewer cognitive resources available for concentration or relaxation. Indeed, in our network, “trouble relaxing” (GAD-3) was closely linked with “feeling irritable” (GAD-5), suggesting a pattern of impaired inhibitory control over stress. This dovetails with evidence that anxiety-related executive dysfunction may mediate the link between anxiety and maladaptive smartphone use in college students. In line with theories that smartphones become an emotional extension of the self (8), the constant attentional pull of phone-related fears could explain why nomophobia’s anxiety symptoms occupy such a central role in the network.

Emotion regulation and dysregulation: Emotion regulation frameworks also shed light on these results. Persistent nomophobia likely leads individuals to rely on their phones as a maladaptive coping strategy to manage negative feelings, a pattern consistent with the “Compensatory Internet Use” and I-PACE (Interaction of Person-Affect-Cognition-Execution) models (48) of problematic technology use. When these individuals are separated from their phones, they may experience intense surges of negative affect without effective coping strategies. The prominence of irritability (agitation) and restlessness among the symptoms suggests emotional dysregulation, wherein anxious arousal spills over into anger and agitation. According to Gross’s process model of emotion regulation (49), difficulty in down-regulating strong emotions can amplify both anxiety and depression. For instance, a nomophobic student who cannot calm themselves when phone access is cut off may become increasingly frustrated and agitated (reflected by GAD-5, “irritable/restless”), which in turn can spiral into pessimistic thoughts, hopelessness, or self-critical rumination, feeding depressive symptoms. The identification of suicidal ideation (PHQ-9) as a particularly potent aggravator in our simulations underscores the extreme of this dysregulation spectrum: when coping resources are completely overwhelmed, thoughts of self-harm emerge. Clinically, this finding highlights that suicidal cognition is not merely a byproduct of depression in such contexts but an active driver of the entire symptom network’s activation. This emphasizes the need for vigilant assessment and intervention on suicidal thinking in nomophobic youth, as it can rapidly escalate the overall psychopathological burden.

Cognitive-affective interplay: From a cognitive-affective perspective, our findings illustrate how negative thought patterns interface with mood symptoms in the nomophobia context. Classical cognitive models of depression posit that persistent negative appraisals and catastrophic thinking fuel low mood, while cognitive models of anxiety emphasize an attentional bias toward perceived threat. In our network, the bridge symptoms GAD-7 (expressing fear of something awful happening) and GAD-6 (excessive fear/afraid feelings) epitomize such catastrophic appraisals. These anxiety symptoms overlap with the general distress component of depression, supporting the idea from Clark and Watson’s tripartite model (50) that a shared factor of negative affect underlies both disorders. At the same time, the tripartite model holds that physiological hyperarousal is specific to anxiety and anhedonia (loss of pleasure) to depression. The high centrality of psychomotor disturbance (PHQ-8) in our results may reflect this physiological arousal aspect: this symptom (which includes agitation or retardation) was strongly connected to both anxious arousal and depressive inertia, potentially serving as a bridge between the physical restlessness of anxiety and the lethargy of depression. Likewise, classic depressive symptoms such as sad mood and guilt (PHQ-2 and PHQ-6) were linked via the aforementioned anxiety bridge symptoms, indicating that general negative affect weaves through both symptom domains. In sum, this cognitive-affective interplay suggests a vicious cycle in which anxious fear about losing phone connection (a form of cognitive hypervigilance) feeds into a broad state of distress, which in turn amplifies depressed mood and fatigue. This perspective helps explain why targeting a general arousal symptom, such as fatigue (PHQ-4), had such widespread simulated effects: improving energy and reducing exhaustion may disrupt the cascade of negative thoughts and feelings that propagate through the network.

Symptom-level comorbidity model: The present study also contributes to theoretical models of comorbidity by emphasizing direct symptom-to-symptom linkages as mechanisms for co-occurrence. Rather than viewing anxiety and depression as entirely separate latent disorders that happen to correlate, our network findings align with contemporary network theory in which comorbidity arises from specific symptoms of one condition directly activating symptoms of another (38). In this framework, nomophobia can be conceptualized as an external stressor that triggers a connected “web” of stress responses across anxiety and mood symptomatology. The bridge nodes we identified (GAD-6 and GAD-7) represent tangible pathways through which anxiety symptoms (like fear of disconnection or feeling something awful might happen) can spark or exacerbate depressive symptoms (such as sleep disturbances, hopelessness, or low mood). For example, an anxious thought about being unable to reach one’s social network could lead to insomnia or restless sleep, which then contributes to fatigue and feelings of hopelessness the next day, illustrating how a chain of symptoms links anxiety with depression. This symptom-driven account of comorbidity explains why certain specific symptoms, rather than whole diagnostic syndromes, act as the critical conduits of overlap between conditions. Our findings refine the network theory of psychopathology by providing evidence in a novel context (college students with high smartphone dependence) that pinpointing and targeting these bridge symptoms might effectively “break the cycle” of nomophobia-related comorbidity. Interventions aimed at the bridge symptoms could sever the reinforcing connections between anxiety and depression clusters, thereby reducing overall distress more efficiently than disorder-level treatments.

## 5 Conclusions

In conclusion, this symptom-oriented network analysis offers a fine-grained view of how depression and anxiety symptoms interlock in the context of nomophobia. Our results highlight that targeting symptoms like low energy or excessive worry in nomophobic students may yield “ripple effects” that alleviate a wider cluster of associated problems. Such a network-informed approach moves beyond treating diagnoses in isolation, instead focusing on strategic symptom interventions that could improve mental health outcomes in the smartphone age. By pinpointing what to target first in the anxiety-depression comorbidity seen with nomophobia, our study lays the groundwork for more efficient prevention and intervention efforts that align with the realities of adolescents’ digital lives.

## 6 Implications

Our network insights suggest several targeted intervention strategies that could be beneficial in clinical and educational settings for adolescents and young adults with nomophobia. First, fatigue/low energy (PHQ-4) emerged as an optimal target for alleviation in the simulations, suggesting that improving sleep and energy levels may have broad mental health benefits in this population. Interventions such as sleep hygiene programs, regular exercise, or cognitive-behavioral therapy for insomnia could help reduce chronic fatigue. Alleviating fatigue in nomophobic students may not only boost mood and motivation but also lower anxiety by reducing overall physiological arousal. In practical terms, helping students establish better sleep routines and rest from constant phone engagement could dampen the entire network of symptoms.

Second, the difficulty in relaxing (GAD-3) and the presence of restlessness/irritability (GAD-5) indicate a need for training in stress management and relaxation techniques. Clinicians might incorporate mindfulness-based stress reduction, breathing exercises, or progressive muscle relaxation into interventions for smartphone-related anxiety. Teaching nomophobic individuals how to deliberately relax and self-soothe without immediately turning to their phones could directly target these symptoms, thereby dampening the cascade that leads from anxiety into other problems. These symptom-focused strategies align with broader therapeutic models that prioritize transdiagnostic skills (e.g., emotional regulation, distress tolerance) to address multiple interlinked symptoms at once.

Another critical implication is the heightened role of suicidal ideation (PHQ-9) as an aggravating node. This finding adds urgency to suicide prevention efforts within this demographic. Campus counselors and mental health practitioners should regularly screen for suicidal thoughts in students who exhibit high nomophobia alongside depression and anxiety symptoms. Evidence that activating suicidal ideation can dramatically amplify the whole network suggests that even subclinical self-harm thoughts should be taken very seriously in this context. Brief cognitive-behavioral interventions that specifically challenge catastrophic beliefs, whether fears about being disconnected from one’s phone or hopeless thoughts about one’s future, might simultaneously reduce anxiety and mitigate suicidal thinking. Psychoeducation about healthy smartphone use is also pertinent: encouraging practices such as “phone curfews” (designating tech-free periods, especially before bed) or scheduled “digital detox” intervals could help nomophobic students gradually reduce their fear of not having immediate phone access. Embedding such digital wellness programs into university health curricula or counseling services could prevent anxiety and depressive symptoms from escalating to crisis levels.

In educational settings, promoting digital balance should be a complementary strategy. Educators and school administrators can acknowledge students’ concerns about their phones and work to address them in healthy ways. For example, teachers might allow short, structured phone-check breaks during long classes, or conversely, incorporate phone-free activities that gently expose students to being without their devices for manageable periods. Such approaches could reduce the ambient anxiety some students feel about missing out or being unreachable. Workshops on emotion regulation and coping skills, offered through school counseling centers or health classes, would further equip students to manage feelings of irritability (related to GAD-5) and fear of negative outcomes (related to GAD-6/GAD-7) without relying on constant connectivity. Given the emerging links between nomophobia and academic burnout (e.g., excessive phone use contributing to sleep loss and stress), universities and high schools should also consider incorporating sleep and stress management training into student orientation programs. By targeting the specific symptom vulnerabilities illuminated by our network (fatigue, relaxation difficulty, irritability, and fear of disconnection), such clinical and educational interventions can be more precisely tailored to break the vicious cycle of phone-related anxiety and depressed mood.

## 7 Limitations

Nonetheless, several important limitations must be acknowledged. First, the study was cross-sectional, which limits any causal interpretations. We discussed bridge and central symptoms in dynamic terms (e.g., as drivers of other symptoms), but our data only capture associations at one point in time. It remains unproven that activating or alleviating a symptom will truly cause downstream changes in other symptoms; longitudinal or experimental data are needed to confirm these hypothesized causal pathways. Second, we dichotomized symptom scores to fit the Ising model (treating each symptom as present vs. absent). While this binary approach simplifies the network estimation and aligns with a screening perspective, it sacrifices information about symptom severity, which could affect the accuracy of edge weights and centrality metrics. Important nuances (for example, moderate vs. extreme levels of a symptom) might be lost in our binary representation. Third, the sample was drawn from a specific population, namely Chinese university students recruited via an online platform, which may limit the generalizability of the findings. This convenience sampling could introduce bias; for instance, students who are more tech-savvy or frequently online might have been more likely to participate, potentially inflating the levels of nomophobia. Moreover, cultural factors unique to China (such as intense academic pressure, family expectations, or differing norms around technology use and mental health stigma) might influence both nomophobia and its psychological correlates in ways that do not perfectly generalize to Western or other cultural contexts. The strong emphasis on academic achievement in China, for example, might exacerbate smartphone-related stress (e.g., constant connectivity to manage studies or social expectations), possibly magnifying the links we observed between attentional strain and mood symptoms. Fourth, although the cutoff for mild nomophobia (average item score > 1) was derived from the original NMP-Q scoring framework, it is relatively inclusive and may have limited the specificity of the sample. Accordingly, our findings are better understood as reflecting symptom associations among adolescents with at least mild nomophobia-related experiences, rather than among a clinically diagnosed nomophobia population. Moreover, in the absence of external diagnostic or validation data, we could not further test the sensitivity of this inclusion criterion. Future studies should assess whether the network structure and simulation results remain stable when more stringent cutoff criteria or independent validation standards are applied. Fifth, there are assumptions inherent in our NIRA simulation approach. The simulation treats each symptom as if it could be “tuned” up or down in isolation, assuming that the estimated network connections are accurate representations of causal links. In reality, interventions on symptoms do not occur in a vacuum, treating one symptom might have side effects or be influenced by external context, and symptoms outside our measured network (such as other unmeasured behaviors or environmental factors) could feed back into the system. Additionally, the network structure we estimated is based on correlational data; even though we used bootstrap techniques to ensure stability of the network, the connections could still reflect unmeasured third variables or confounding factors. This discrepancy is consistent with prior warnings that centrality should not be conflated with intervention priority (44, 45). It highlights the need for caution when interpreting cross-sectional network maps: a symptom that appears structurally important (high centrality) is not guaranteed to be the most impactful one to treat. Overall, while our approach offers promising insights, any network-derived intervention suggestions should be validated with controlled studies before clinical adoption.

## 8 Future directions

Our findings open several avenues for future research. Longitudinal studies are essential to establish temporal and causal relationships in the nomophobia-anxiety-depression network. A multi-wave design or intensive time-series data (e.g., ecological momentary assessments) could determine whether certain symptoms reliably precede and predict changes in others. For instance, do spikes in worry or fear about disconnection (anxiety symptoms) reliably precede subsequent increases in fatigue or sadness the next day? Ambulatory assessment of daily smartphone use and mood could help verify if heavy phone dependence on a given day leads to next-day emotional exhaustion and depressive affect. Demonstrating such time-ordered patterns would strengthen confidence in the causal interpretations of our network (e.g., that reducing worry would indeed lead to improved mood, or that increased phone-related anxiety leads to immediate sleep disturbances).

Experimental intervention studies will also be crucial to validate the targets suggested by our simulation. Randomized controlled trials could test whether intervening on the identified symptoms yields the expected ripple effects on the network. For example, one could design an intervention to specifically improve energy and reduce fatigue (PHQ-4), such as a program to extend sleep duration or improve sleep quality, and then observe whether participants receiving this intervention show broader reductions in other depressive and anxious symptoms compared to a control group. Similarly, a targeted cognitive-behavioral intervention could focus on challenging the catastrophic thoughts central to GAD-6 and GAD-7 (the fear of awful events and feeling afraid without the phone). If such an intervention not only reduces anxiety but also yields secondary improvements in depressive symptoms, it would empirically support the idea that those bridge anxiety symptoms maintain the comorbidity. Another important experiment would be to see if directly reducing suicidal ideation, for instance, via a brief safety planning or crisis intervention for those with mild suicidal thoughts, disproportionately decreases the overall symptom network activation in nomophobic individuals. Ethically, this is a critical question for prevention: addressing suicidality early might forestall a cascade of worsening across the system.

Beyond self-reported symptoms, future research should integrate objective behavioral and physiological data to enrich the network model. Smartphone usage logs (e.g., total screen time, frequency of checking the phone, durations without access) could be combined with symptom reports to see how real-life behavior correlates with the symptom network structure. For example, one could test if students who exhibit the predicted network pattern (e.g., high worry leading to high fatigue) actually have phone usage patterns (like midnight phone use leading to poor sleep) that correspond to those symptoms. Wearable devices that track sleep continuity or heart rate variability (HRV) could provide physiological markers of arousal and stress to complement self-reported irritability and anxiety. Incorporating such data might align the psychological symptom network with biological markers, offering a more holistic picture of nomophobia’s impact. Additionally, comparative studies across different populations would determine the generality of our findings. It would be informative to examine whether the symptom network of nomophobia with anxiety/depression looks similar in Western college students or in younger adolescents and high school students. Cultural and developmental factors could alter which symptoms become central or bridging; understanding these differences could tailor interventions to specific groups. Ultimately, multi-method and longitudinal research will be key to translating our cross-sectional network insights into robust, evidence-based intervention strategies. 

## Data Availability

The original contributions presented in the study are included in the article/**Supplementary Material**. Further inquiries can be directed to the corresponding authors.
