# Supplementary material for: Exploring which symptom should be targeted first in the comorbidity of anxiety and depression among adolescents with nomophobia: insight from a simulation network analysis
Source: Front Psychiatry. 2026 Apr 13;17:1772083. doi: 10.3389/fpsyt.2026.1772083 (PMC13114011; doi:10.3389/fpsyt.2026.1772083)
Supplement: Supplementary file 1 [file SupplementaryFile1.docx]

**Online Supplemental Materials**

**Tables**

**Table S1: Descriptive Statistics and Differences between Groups.**

| **Variable** |  | **Nomophobia** | | | |  | **Depression** | | | |  | **Anxiety** | | | |
| --- | --- | --- | --- | --- | --- | --- | --- | --- | --- | --- | --- | --- | --- | --- | --- |
|  |  | ***M*** | ***SD*** | ***p*** | ***Cohen’s d*** |  | ***M*** | ***SD*** | ***p*** | ***Cohen’s d*** |  | ***M*** | ***SD*** | ***p*** | ***Cohen’s d*** |
| **Gender** |  |  |  |  |  |  |  |  |  |  |  |  |  |  |  |
| Male |  | 3.15 | 1.25 | < .001 | -0.26 |  | 5.30 | 4.71 | .210 | 0.07 |  | 6.35 | 5.59 | < .001 | 0.18 |
| Female |  | 3.49 | 1.32 |  |  |  | 5.01 | 4.10 |  |  |  | 5.42 | 4.80 |  |  |
| **Only child** |  |  |  |  |  |  |  |  |  |  |  |  |  |  |  |
| Yes |  | 3.31 | 1.32 | .460 | -0.05 |  | 4.79 | 4.34 | .147 | -0.09 |  | 5.38 | 5.03 | .140 | -0.09 |
| No |  | 3.38 | 1.30 |  |  |  | 5.19 | 4.33 |  |  |  | 5.85 | 5.14 |  |  |

***Note.*** *M*, mean. *SD*, standard deviation. Welch’s tests were performed for all variables due to violations of the assumption of equal variances.

**Table S2: Descriptive Statistics and Correlation Matrix between Variables.**

| **Variable** | ***M*** | ***SD*** | **1** | **2** | **3** | **4** | **5** | **6** | **7** |
| --- | --- | --- | --- | --- | --- | --- | --- | --- | --- |
| 1. Gender | 1.63 | 0.48 | — |  |  |  |  |  |  |
| 2. Age | 5.78 | 1.60 | –.05 | — |  |  |  |  |  |
| 3. Only child | 1.81 | 0.39 | .15^***^ | .01 | — |  |  |  |  |
| 4. Parental marriage | 1.35 | 0.94 | –.02 | –.02 | –.07^**^ | — |  |  |  |
| 5. Parental relationship | 1.54 | 0.59 | .08^**^ | –.03 | –.02 | .17^***^ | — |  |  |
| 6. Nomophobia | 3.36 | 1.31 | .12^***^ | –.15^***^ | .02 | .05 | .17^***^ | — |  |
| 7. Anxiety | 5.11 | 4.33 | –.03 | –.08^***^ | .04 | .09^***^ | .20^***^ | .39^***^ | — |
| 8. Depression | 5.76 | 5.12 | –.09^***^ | –.07^**^ | .04 | .12^***^ | .21^***^ | .36^***^ | .74^***^ |

*Note*. ** *p* < .01. *** *p* < .001.

**Table S3: The Edge-Weight Matrix, Thresholds, and Standardized Centrality in the Depression Network.**

| Node |  | **Edge-weight matrix** | | | | | | | | |  | **Thresholds** | |  | **Standardized**  **EI Centrality** | |
| --- | --- | --- | --- | --- | --- | --- | --- | --- | --- | --- | --- | --- | --- | --- | --- | --- |
|  |  | PHQ1 | PHQ2 | PHQ3 | PHQ4 | PHQ5 | PHQ6 | PHQ7 | PHQ8 | PHQ9 |  |  |  |  |  |  |
| PHQ1: Anhedonia |  | 0 | 1.225 | 0.857 | 0.913 | 0.437 | 0.420 | 0.722 | 0.296 | 0 |  | -1.502 |  |  | -1.237 |  |
| PHQ2: Depressed or sad mood |  | 1.225 | 0 | 0.448 | 1.106 | 0.284 | 1.023 | 0.178 | 0.787 | 1.031 |  | -3.237 |  |  | 0.204 |  |
| PHQ3: Sleep difficulties |  | 0.857 | 0.448 | 0 | 1.323 | 0.971 | 0.318 | 0.341 | 0.262 | 0.556 |  | -2.539 |  |  | -0.993 |  |
| PHQ4: Fatigue |  | 0.913 | 1.106 | 1.323 | 0 | 1.160 | 0.392 | 0.506 | 0.667 | 0 |  | -1.923 |  |  | 0.185 |  |
| PHQ5: Appetite changes |  | 0.437 | 0.284 | 0.971 | 1.160 | 0 | 0.342 | 0.914 | 0.838 | 0 |  | -2.146 |  |  | -1.147 |  |
| PHQ6: Guilt |  | 0.420 | 1.023 | 0.318 | 0.392 | 0.342 | 0 | 1.232 | 1.168 | 1.582 |  | -3.181 |  |  | 0.675 |  |
| PHQ7: Concentration difficulties |  | 0.722 | 0.178 | 0.341 | 0.506 | 0.914 | 1.232 | 0 | 1.655 | 0.895 |  | -2.398 |  |  | 0.634 |  |
| PHQ8: Motor disturbances |  | 0.296 | 0.787 | 0.262 | 0.667 | 0.838 | 1.168 | 1.655 | 0 | 1.747 |  | -4.762 |  |  | 1.795 |  |
| PHQ9: Suicide ideation |  | 0 | 1.031 | 0.556 | 0 | 0 | 1.582 | 0.895 | 1.747 | 0 |  | -4.593 |  |  | -0.117 |  |

***Note*.** EI, Expected Influence.

**Table S4: The Edge-Weight Matrix, Thresholds, and Standardized Centrality in the Anxiety Network.**

| Node |  | **Edge-weight matrix** | | | | | | |  | **Thresholds** | |  | **Standardized**  **EI Centrality** | |
| --- | --- | --- | --- | --- | --- | --- | --- | --- | --- | --- | --- | --- | --- | --- |
|  |  | GAD1 | GAD2 | GAD3 | GAD4 | GAD5 | GAD6 | GAD7 |  |  |  |  |  |  |
| GAD1: Anxiousness |  | 0 | 1.998 | 0.911 | 0.275 | 0.939 | 1.063 | 0.156 |  | -1.746 |  |  | -0.997 |  |
| GAD2: Uncontrollable worry |  | 1.998 | 0 | 1.290 | 1.189 | 0.595 | 0.200 | 0.818 |  | -3.251 |  |  | 0.271 |  |
| GAD3: Excessive worry |  | 0.911 | 1.290 | 0 | 1.034 | 1.057 | 0.518 | 1.230 |  | -1.853 |  |  | 0.187 |  |
| GAD4: Trouble relaxing |  | 0.275 | 1.189 | 1.034 | 0 | 1.680 | 0.957 | 0.580 |  | -2.772 |  |  | -0.365 |  |
| GAD5: Restlessness |  | 0.939 | 0.595 | 1.057 | 1.680 | 0 | 1.062 | 1.713 |  | -4.585 |  |  | 1.892 |  |
| GAD6: Irritability |  | 1.063 | 0.200 | 0.518 | 0.957 | 1.062 | 0 | 1.490 |  | -2.136 |  |  | -1.084 |  |
| GAD7: Feeling afraid |  | 0.156 | 0.818 | 1.230 | 0.580 | 1.713 | 1.490 | 0 |  | -3.728 |  |  | 0.096 |  |

***Note*.** EI, Expected Influence.

**Table S5: The Edge-Weight Matrix of the Depression Network based on the Continuous Data.**

| Node |  | **Edge-weight matrix** | | | | | | | | |  |
| --- | --- | --- | --- | --- | --- | --- | --- | --- | --- | --- | --- |
|  |  | PHQ1 | PHQ2 | PHQ3 | PHQ4 | PHQ5 | PHQ6 | PHQ7 | PHQ8 | PHQ9 |  |
| PHQ1: Anhedonia |  | 0 | 0.219 | 0.129 | 0.151 | 0.083 | 0.088 | 0.107 | 0 | 0 |  |
| PHQ2: Depressed or sad mood |  | 0.219 | 0 | 0.061 | 0.194 | 0 | 0.168 | 0.073 | 0.116 | 0.197 |  |
| PHQ3: Sleep difficulties |  | 0.129 | 0.061 | 0 | 0.260 | 0.183 | 0 | 0.066 | 0 | 0 |  |
| PHQ4: Fatigue |  | 0.151 | 0.194 | 0.260 | 0 | 0.228 | 0 | 0.090 | 0.058 | 0 |  |
| PHQ5: Appetite changes |  | 0.083 | 0 | 0.183 | 0.228 | 0 | 0.093 | 0.180 | 0.107 | 0 |  |
| PHQ6: Guilt |  | 0.088 | 0.168 | 0 | 0 | 0.093 | 0 | 0.225 | 0.215 | 0.156 |  |
| PHQ7: Concentration difficulties |  | 0.107 | 0.073 | 0.066 | 0.090 | 0.180 | 0.225 | 0 | 0.187 | 0 |  |
| PHQ8: Motor disturbances |  | 0 | 0.116 | 0 | 0.058 | 0.107 | 0.215 | 0.187 | 0 | 0.290 |  |
| PHQ9: Suicide ideation |  | 0 | 0.197 | 0 | 0 | 0 | 0.156 | 0 | 0.290 | 0 |  |

**Table S6: The Edge-Weight Matrix of the Anxiety Network based on the Continuous Data.**

| Node |  | **Edge-weight matrix** | | | | | | |
| --- | --- | --- | --- | --- | --- | --- | --- | --- |
|  |  | GAD1 | GAD2 | GAD3 | GAD4 | GAD5 | GAD6 | GAD7 |
| GAD1: Anxiousness |  | 0 | 0.307 | 0.230 | 0.048 | 0.119 | 0.175 | 0 |
| GAD2: Uncontrollable worry |  | 0.307 | 0 | 0.233 | 0.219 | 0.066 | 0.051 | 0.112 |
| GAD3: Excessive worry |  | 0.230 | 0.233 | 0 | 0.219 | 0 | 0.057 | 0.146 |
| GAD4: Trouble relaxing |  | 0.048 | 0.219 | 0.219 | 0 | 0.219 | 0.230 | 0.059 |
| GAD5: Restlessness |  | 0.119 | 0.066 | 0 | 0.219 | 0 | 0.133 | 0.368 |
| GAD6: Irritability |  | 0.175 | 0.051 | 0.057 | 0.230 | 0.133 | 0 | 0.215 |
| GAD7: Feeling afraid |  | 0 | 0.112 | 0.146 | 0.059 | 0.368 | 0.215 | 0 |

**Table S7: The Edge-Weight Matrix, Thresholds, and Standardized Centrality in the Depression-Anxiety Network.**

| **Node** |  | **Edge-weight matrix** | | | | | | | | | | | | | | | |  | **Thresholds** | |  | **Standardized Centrality** | | | |
| --- | --- | --- | --- | --- | --- | --- | --- | --- | --- | --- | --- | --- | --- | --- | --- | --- | --- | --- | --- | --- | --- | --- | --- | --- | --- |
|  |  | PHQ1 | PHQ2 | PHQ3 | PHQ4 | PHQ5 | PHQ6 | PHQ7 | PHQ8 | PHQ9 | GAD1 | GAD2 | GAD3 | GAD4 | GAD5 | GAD6 | GAD7 |  |  |  |  | EI | | Bridge EI | |
| **Depression** |  |  |  |  |  |  |  |  |  |  |  |  |  |  |  |  |  |  |  |  |  |  |  |  |  |
| PHQ1 |  | 0 | 1.116 | 0.815 | 0.851 | 0.415 | 0.320 | 0.650 | 0.198 | 0 | 0 | 0 | 0 | 0 | 0.388 | 0.238 | 0 |  | -1.513 |  |  | -1.509 |  | -1.280 |  |
| PHQ2 |  | 1.116 | 0 | 0.319 | 0.995 | 0.213 | 0.822 | 0.095 | 0.568 | 0.801 | 0 | 0.216 | 0 | 0.268 | 0.239 | 0.190 | 0.469 |  | -3.577 |  |  | 0.232 |  | 0.749 |  |
| PHQ3 |  | 0.815 | 0.319 | 0 | 1.217 | 0.924 | 0.220 | 0.285 | 0.138 | 0.447 | 0.210 | 0.041 | 0.129 | 0.352 | 0 | 0 | 0 |  | -2.806 |  |  | -1.371 |  | -0.997 |  |
| PHQ4 |  | 0.851 | 0.995 | 1.217 | 0 | 1.086 | 0.224 | 0.418 | 0.523 | 0 | 0.268 | 0.254 | 0.289 | 0 | 0 | 0.378 | 0 |  | -2.146 |  |  | 0.485 |  | 0.228 |  |
| PHQ5 |  | 0.415 | 0.213 | 0.924 | 1.086 | 0 | 0.268 | 0.861 | 0.754 | 0 | 0.206 | 0.199 | 0 | 0.050 | 0 | 0.059 | 0 |  | -2.336 |  |  | -1.452 |  | -1.581 |  |
| PHQ6 |  | 0.320 | 0.822 | 0.220 | 0.224 | 0.268 | 0 | 1.113 | 1.005 | 1.417 | 0 | 0.070 | 0.388 | 0 | 0 | 0.486 | 0.223 |  | -3.497 |  |  | 0.556 |  | 0.173 |  |
| PHQ7 |  | 0.650 | 0.095 | 0.285 | 0.418 | 0.861 | 1.113 | 0 | 1.511 | 0.701 | 0.148 | 0.056 | 0 | 0 | 0.037 | 0.309 | 0 |  | -2.452 |  |  | 0.063 |  | -1.484 |  |
| PHQ8 |  | 0.198 | 0.568 | 0.138 | 0.523 | 0.754 | 1.005 | 1.511 | 0 | 1.599 | 0.233 | 0.236 | 0 | 0 | 0.532 | 0 | 0.381 |  | -5.134 |  |  | 2.036 |  | 0.747 |  |
| PHQ9 |  | 0 | 0.801 | 0.447 | 0 | 0 | 1.417 | 0.701 | 1.599 | 0 | 0 | 0 | 0 | 0.436 | 0.206 | 0 | 0.642 |  | -4.897 |  |  | 0.151 |  | 0.485 |  |
| **Anxiety** |  |  |  |  |  |  |  |  |  |  |  |  |  |  |  |  |  |  |  |  |  |  |  |  |  |
| GAD1 |  | 0 | 0 | 0.210 | 0.268 | 0.206 | 0 | 0.148 | 0.233 | 0 | 0 | 1.838 | 0.834 | 0.143 | 0.789 | 0.863 | 0 |  | -1.886 |  |  | -0.796 |  | -0.104 |  |
| GAD2 |  | 0 | 0.216 | 0.041 | 0.254 | 0.199 | 0.070 | 0.056 | 0.236 | 0 | 1.838 | 0 | 1.201 | 1.043 | 0.489 | 0.046 | 0.655 |  | -3.364 |  |  | 0.276 |  | -0.086 |  |
| GAD3 |  | 0 | 0 | 0.129 | 0.289 | 0 | 0.388 | 0 | 0 | 0 | 0.834 | 1.201 | 0 | 0.910 | 0.983 | 0.391 | 1.070 |  | -1.944 |  |  | 0.080 |  | -0.796 |  |
| GAD4 |  | 0 | 0.268 | 0.352 | 0 | 0.050 | 0 | 0 | 0 | 0.436 | 0.143 | 1.043 | 0.910 | 0 | 1.479 | 0.785 | 0.365 |  | -2.521 |  |  | -0.401 |  | 0.005 |  |
| GAD5 |  | 0.388 | 0.239 | 0 | 0 | 0 | 0 | 0.037 | 0.532 | 0.206 | 0.789 | 0.489 | 0.983 | 1.479 | 0 | 0.860 | 1.393 |  | -4.717 |  |  | 1.666 |  | 0.804 |  |
| GAD6 |  | 0.238 | 0.190 | 0 | 0.378 | 0.059 | 0.486 | 0.309 | 0 | 0 | 0.863 | 0.046 | 0.391 | 0.785 | 0.860 | 0 | 1.226 |  | -2.468 |  |  | -0.401 |  | 1.493 |  |
| GAD7 |  | 0 | 0.469 | 0 | 0 | 0 | 0.223 | 0 | 0.381 | 0.642 | 0 | 0.655 | 1.070 | 0.365 | 1.393 | 1.226 | 0 |  | -3.324 |  |  | 0.384 |  | 1.644 |  |

***Note*.** EI, Expected Influence. Bridge EI, bridge Expected Influence.

**Table S8: The Edge-Weight Matrix of the Depression-Anxiety Network based on the Continuous Data.**

| **Node** |  | **Edge-weight matrix** | | | | | | | | | | | | | | | |
| --- | --- | --- | --- | --- | --- | --- | --- | --- | --- | --- | --- | --- | --- | --- | --- | --- | --- |
|  |  | PHQ1 | PHQ2 | PHQ3 | PHQ4 | PHQ5 | PHQ6 | PHQ7 | PHQ8 | PHQ9 | GAD1 | GAD2 | GAD3 | GAD4 | GAD5 | GAD6 | GAD7 |
| **Depression** |  |  |  |  |  |  |  |  |  |  |  |  |  |  |  |  |  |
| PHQ1 |  | 0 | 0.206 | 0.126 | 0.149 | 0.078 | 0.084 | 0.103 | 0 | 0 | 0 | 0 | 0 | 0 | 0 | 0 | 0 |
| PHQ2 |  | 0.206 | 0 | 0 | 0.166 | 0 | 0.124 | 0.058 | 0.065 | 0.176 | 0 | 0 | 0 | 0 | 0 | 0 | 0.081 |
| PHQ3 |  | 0.126 | 0 | 0 | 0.247 | 0.173 | 0 | 0.059 | 0 | 0 | 0 | 0 | 0 | 0 | 0 | 0 | 0 |
| PHQ4 |  | 0.149 | 0.166 | 0.247 | 0 | 0.212 | 0 | 0.083 | 0 | 0 | 0 | 0 | 0.048 | 0 | 0 | 0 | 0 |
| PHQ5 |  | 0.078 | 0 | 0.173 | 0.212 | 0 | 0.076 | 0.174 | 0.092 | 0 | 0 | 0 | 0 | 0 | 0 | 0 | 0 |
| PHQ6 |  | 0.084 | 0.124 | 0 | 0 | 0.076 | 0 | 0.216 | 0.185 | 0.146 | 0 | 0 | 0.078 | 0 | 0 | 0 | 0.066 |
| PHQ7 |  | 0.103 | 0.058 | 0.059 | 0.083 | 0.174 | 0.216 | 0 | 0.170 | 0 | 0 | 0 | 0 | 0 | 0 | 0 | 0 |
| PHQ8 |  | 0 | 0.065 | 0 | 0 | 0.092 | 0.185 | 0.170 | 0 | 0.266 | 0 | 0 | -0.057 | 0 | 0.103 | 0.057 | 0.067 |
| PHQ9 |  | 0 | 0.176 | 0 | 0 | 0 | 0.146 | 0 | 0.266 | 0 | 0 | 0 | 0 | 0 | 0 | 0 | 0 |
| **Anxiety** |  |  |  |  |  |  |  |  |  |  |  |  |  |  |  |  |  |
| GAD1 |  | 0 | 0 | 0 | 0 | 0 | 0 | 0 | 0 | 0 | 0 | 0.294 | 0.217 | 0 | 0.095 | 0.148 | 0 |
| GAD2 |  | 0 | 0 | 0 | 0 | 0 | 0 | 0 | 0 | 0 | 0.294 | 0 | 0.230 | 0.211 | 0.052 | 0 | 0.092 |
| GAD3 |  | 0 | 0 | 0 | 0.048 | 0 | 0.078 | 0 | -0.057 | 0 | 0.217 | 0.230 | 0 | 0.213 | 0 | 0.048 | 0.138 |
| GAD4 |  | 0 | 0 | 0 | 0 | 0 | 0 | 0 | 0 | 0 | 0 | 0.211 | 0.213 | 0 | 0.200 | 0.210 | 0.045 |
| GAD5 |  | 0 | 0 | 0 | 0 | 0 | 0 | 0 | 0.103 | 0 | 0.095 | 0.052 | 0 | 0.200 | 0 | 0.096 | 0.317 |
| GAD6 |  | 0 | 0 | 0 | 0 | 0 | 0 | 0 | 0.057 | 0 | 0.148 | 0 | 0.048 | 0.210 | 0.096 | 0 | 0.180 |
| GAD7 |  | 0 | 0.081 | 0 | 0 | 0 | 0.066 | 0 | 0.067 | 0 | 0 | 0.092 | 0.138 | 0.045 | 0.317 | 0.180 | 0 |

**Table S9: Simulated Alleviating and Aggravating Interventions for Three Networks.**

| **Symptoms in Network** | **Original**  **Sum score** | **Alleviating Intervention** | |  | **Aggravating Intervention** | |
| --- | --- | --- | --- | --- | --- | --- |
|  |  | **Sum score** | **NIRA** |  | **Sum score** | **NIRA** |
| **Depression Network** |  |  |  |  |  |  |
| PHQ1: Anhedonia | 4.84 | 2.27 | 2.57 |  | 6.25 | 1.41 |
| PHQ2: Depressed or sad mood | 4.84 | 2.66 | 2.18 |  | 6.94 | 2.10 |
| PHQ3: Sleep difficulties | 4.84 | 2.61 | 2.22 |  | 6.75 | 1.91 |
| PHQ4: Fatigue | 4.84 | 2.14 | **2.70** |  | 6.35 | 1.52 |
| PHQ5: Appetite changes | 4.84 | 2.46 | 2.37 |  | 6.60 | 1.76 |
| PHQ6: Guilt | 4.84 | 2.76 | 2.08 |  | 7.11 | 2.28 |
| PHQ7: Concentration difficulties | 4.84 | 2.36 | 2.48 |  | 6.75 | 1.91 |
| PHQ8: Motor disturbances | 4.84 | 2.96 | 1.88 |  | 7.33 | 2.49 |
| PHQ9: Suicide ideation | 4.84 | 3.48 | 1.36 |  | 7.36 | **2.52** |
| **Anxiety Network** |  |  |  |  |  |  |
| GAD1: Anxiousness | 4.19 | 1.93 | 2.26 |  | 5.45 | 1.26 |
| GAD2: Uncontrollable worry | 4.19 | 2.09 | 2.10 |  | 5.75 | 1.56 |
| GAD3: Excessive worry | 4.19 | 1.88 | **2.31** |  | 5.52 | 1.33 |
| GAD4: Trouble relaxing | 4.19 | 2.13 | 2.07 |  | 5.74 | 1.55 |
| GAD5: Restlessness | 4.19 | 2.29 | 1.90 |  | 6.00 | **1.80** |
| GAD6: Irritability | 4.19 | 2.11 | 2.09 |  | 5.56 | 1.37 |
| GAD7: Feeling afraid | 4.19 | 2.34 | 1.85 |  | 5.95 | 1.76 |
| **Depression-Anxiety Network** |  |  |  |  |  |  |
| **Depression** |  |  |  |  |  |  |
| PHQ1: Anhedonia | 8.74 | 4.89 | 3.85 |  | 10.92 | 2.18 |
| PHQ2: Depressed or sad mood | 8.74 | 5.50 | 3.23 |  | 12.22 | 3.48 |
| PHQ3: Sleep difficulties | 8.74 | 5.48 | 3.26 |  | 11.68 | 2.94 |
| PHQ4: Fatigue | 8.74 | 4.48 | **4.25** |  | 11.10 | 2.36 |
| PHQ5: Appetite changes | 8.74 | 5.32 | 3.42 |  | 11.42 | 2.68 |
| PHQ6: Guilt | 8.74 | 5.58 | 3.16 |  | 12.27 | 3.54 |
| PHQ7: Concentration difficulties | 8.74 | 5.20 | 3.54 |  | 11.69 | 2.95 |
| PHQ8: Motor disturbances | 8.74 | 5.96 | 2.77 |  | 12.71 | 3.97 |
| PHQ9: Suicide ideation | 8.74 | 6.76 | 1.98 |  | 12.76 | **4.03** |
| **Anxiety** |  |  |  |  |  |  |
| GAD1: Anxiousness | 8.74 | 4.66 | 4.08 |  | 11.08 | 2.34 |
| GAD2: Uncontrollable worry | 8.74 | 4.94 | 3.80 |  | 11.62 | 2.89 |
| GAD3: Excessive worry | 8.74 | 4.45 | **4.28** |  | 10.95 | 2.22 |
| GAD4: Trouble relaxing | 8.74 | 4.79 | 3.95 |  | 11.50 | 2.76 |
| GAD5: Restlessness | 8.74 | 5.22 | 3.52 |  | 12.27 | 3.53 |
| GAD6: Irritability | 8.74 | 4.64 | 4.10 |  | 11.44 | 2.70 |
| GAD7: Feeling afraid | 8.74 | 5.10 | 3.64 |  | 11.94 | 3.21 |

***Note.*** The highest NIRA values in interventions for each network were in bold.

**Figures**


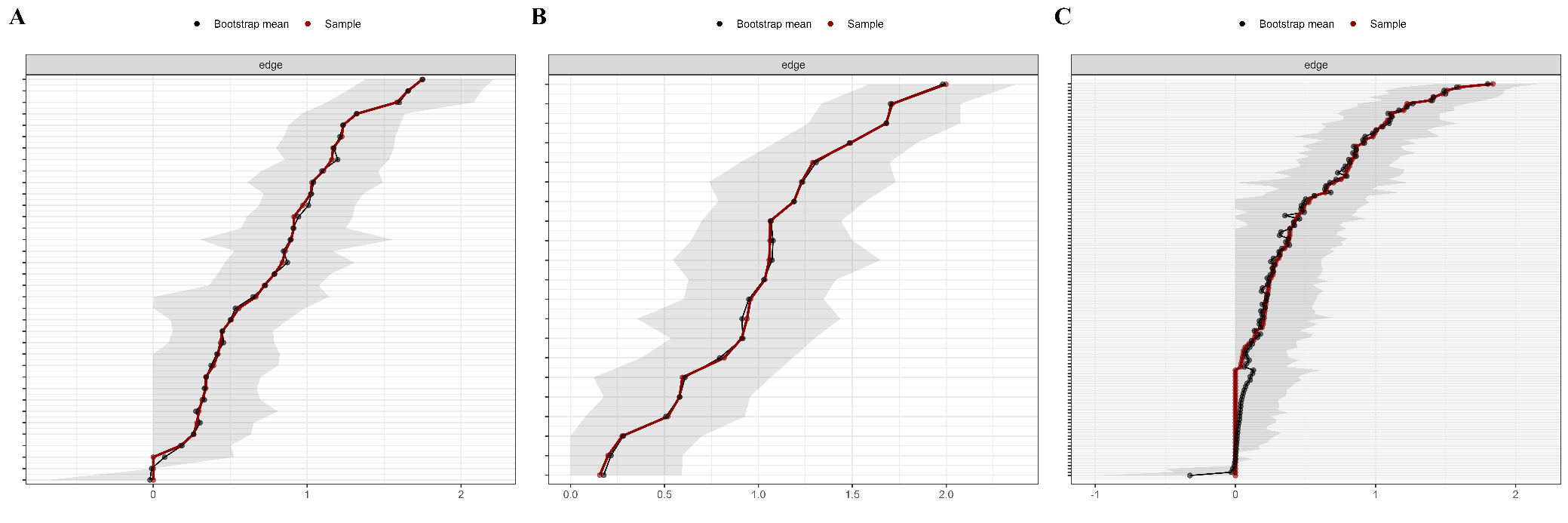


**Figure S1: Bootstrapped Accuracy of Edge Weights in the Depression (A), Anxiety (B), and Comorbidity (C) Networks.**

***Note*.** the red line indicates the estimated edge, and the dark area indicates the 95% bootstrap CI.


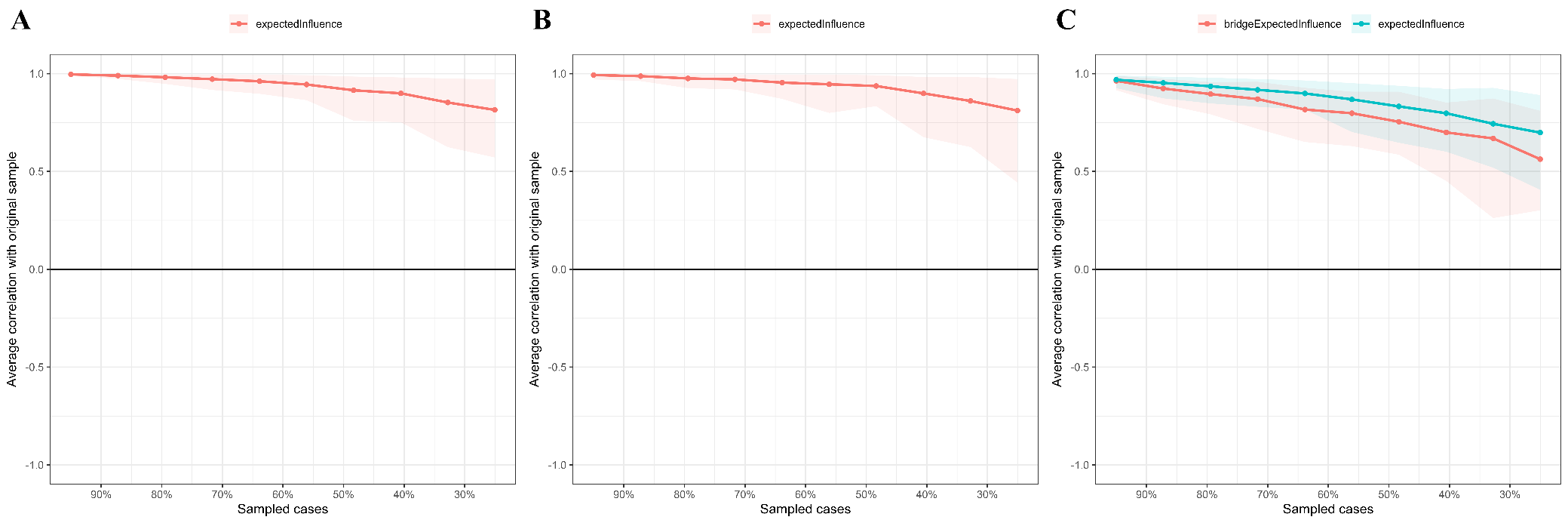


**Figure S2: Bootstrapped Stability of Centrality for the Depression (A), Anxiety (B), and Comorbidity (C) Networks.**

***Note*.** **y-axis**: Mean correlation between centrality metrics from the original network and those from networks re-estimated after progressively removing larger proportions of the sample. Correlation exceeding 0.25, 0.5, and 0.75 reflects acceptable, good, and excellent stability, respectively.





**Figure S3: Bootstrapped Differences in Edge Weights in the Depression (A), Anxiety (B), And Comorbidity (C) Networks.**

***Note*.** Box colors indicate whether edge weights differ significantly: black denotes significant differences, while gray indicates no significant difference. The diagonal line reflects edge strength, with colors ranging from red (negative associations) to white (weaker edges) to blue (stronger positive connections).


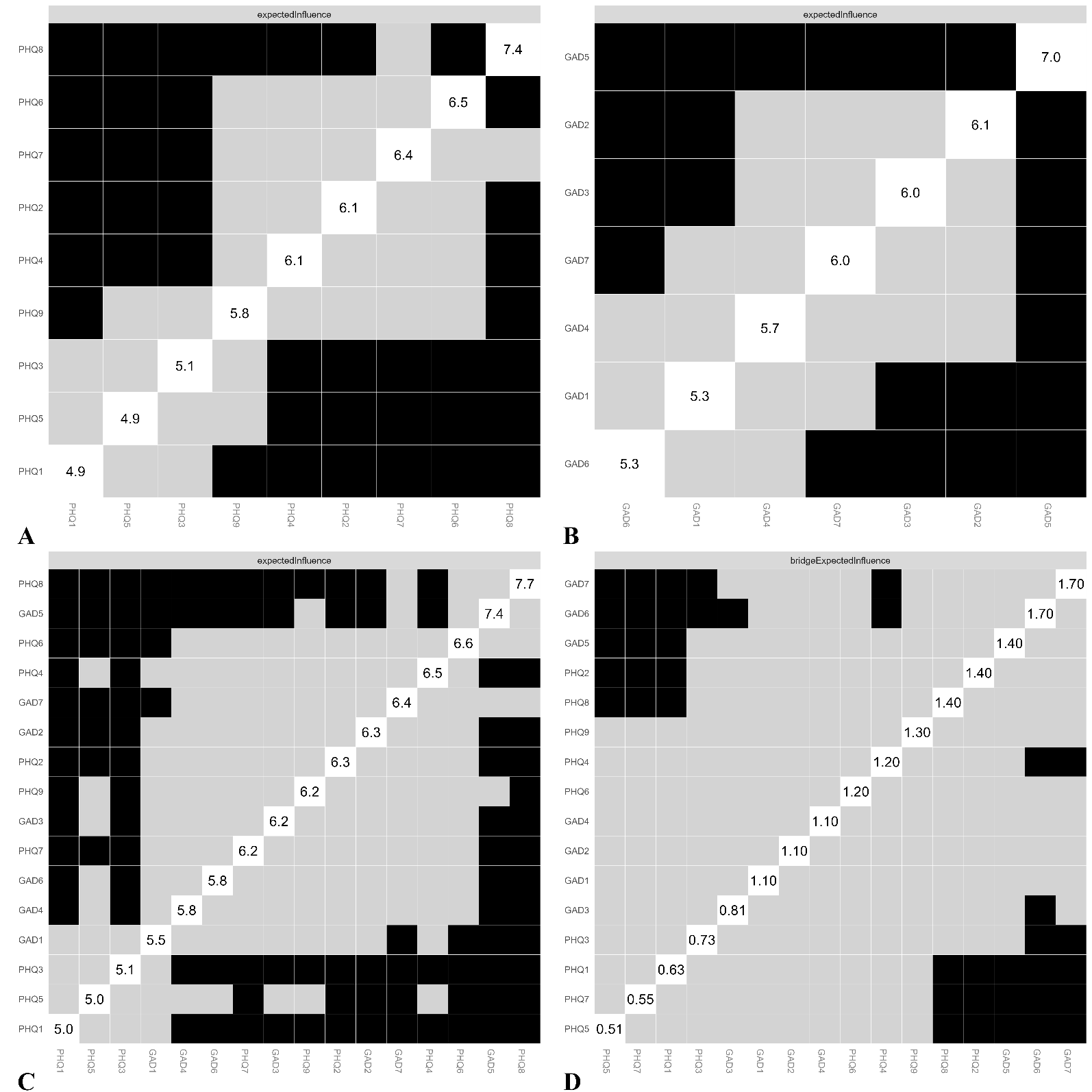


**Figure S4: Bootstrapped Differences in Centrality Values Between Nodes in the Depression (A), Anxiety (B), and Comorbidity (C & D) Networks.**

***Note*.** Box colors indicate whether edge weights differ significantly: black denotes significant differences, while gray indicates no significant difference. The numbers in the white boxes (i.e., diagonal line) represent the values of the nodes’ raw centrality.
